# Diversity in boardroom and debt financing: A case from China

**DOI:** 10.3389/fpsyg.2022.1006293

**Published:** 2022-09-27

**Authors:** Xinbo Sun, Muneeb Ahmad, Kamran Tahir, Hammad Zafar

**Affiliations:** ^1^School of Business Administration, Northeastern University, Shenyang, China; ^2^Lahore School of Accountancy and Finance, Lahore, Pakistan; ^3^Allama Iqbal College of Commerce, Lahore, Pakistan

**Keywords:** debt financing, board diversity, risky decision, board decisions, mass theory

## Abstract

The study aims to explore the role of gender diversity in debt financing choices among Chinese listed firms. The study used the Chinese listed firm's data from 1991 to 2022 from the Chinese Stock Market return. The study used the fixed effect regression analysis and revealed that gender diversity positively affects debt financing among Chinese firms. Additionally, mass theory results suggested that at least three females on the board significantly influence firms. It served as the voice of gender diversity to influence the board's decisions regarding debt financing. The study has several theoretical and practical implications. This study will enlighten the Chinese boardroom dynamics by reassuring them to add more females to diversity policies. It will benefit future studies on boardroom activities and debt financing in emerging economies. It will be practical guidance for the Chinese policymakers, governing authorities, and corporate executives. The study stresses the need for significant diversity on the board rather than one female presence on the board. Secondly, this study contradicts the stereotype perception that females are not making risky decisions.

## Introduction

Board diversity got substantial attention in recent years, and its effects have been explored from different perspectives, such as on research and development (Almor et al., 2019; AlHares, 2020; Xie et al., 2020) and firm performance (Aggarwal et al., 2019; Fernández-Temprano and Tejerina-Gaite, 2020; Ozdemir, 2020). Apart from the direct relationship, few studies took board diversity's indirect effect (Miller and del Carmen Triana, 2009; Rashid, 2020). A significant amount of literature in the psychology field concurs that females are risk averse than men. For instance, through a survey, Jianakoplos and Bernasek (1998) analyzed that female wealth holding is less risky than males. In the same line, Brooks and Zank (2005) conducted an experiment on 49 students to invest in lotteries and found that females are more risk averse. This widespread view that females' risk-averse behavior may be the potential cause less presence of females in the top management, also known as the “glass ceiling.”

Despite the “glass ceiling” view, studies show that females are not always risk averse. Dwyer et al. (2003) documented that females are not risk averse when they are well-informed. They argued that knowledge disparities drive risk-averse labels on females in the literature. In the same line, Johnson and Powell (1994) argued that there is no difference between male and female decision-making in managerial settings. Weber and Zulehner (2010) contradict this view by studying females' and males' perceived risk of new start-ups. Their study found that new start-ups led by females have higher chances of survival than those led by men in entrepreneurship settings. The question that animated at the World Economic Forum in February 2009 that, “What if Lehman Brothers had been Lehman Sisters?” will the world see the same outcome or not? These questions improved the number of female presence in top management due to the belief that the answer to these questions might be affirmative (Liu et al., 2014). Mulcahy and Linehan (2014) found an interesting result that female presence on board is high in firms that are in precarious situations. Thus, females may be more risk-averse in risky environments.

Prior literature supports that females are risk averse and unable to distinguish between risk averse and averse to uncertainty. Adams and Funk (2012) document that female decisions are stakeholder-oriented, making them risk averse. In the same direction, Ali et al. (2014) argued that more presence of females on the board resulted in high employee productivity, which supports the Adams and Funk (2012) findings. Adams and Ferreira (2009) found that female presence on the board resulted in better resource allocation of the firm, which supports the stewardship view. Babalos et al. (2015) found that females prefer investment in growth-oriented stocks over their male directors. Faccio et al. (2016) found that female CEOs took longer to decide on investment opportunities to avoid volatile earnings. This longer time describes the females as risk-averse but may explain their avoidance of ambiguity and uncertainty. These arguments confirm that females are risk sensitive but not risk averse.

Given the view of females with less averse appetites, it is interesting to know whether this stereotypical view exists in a firm's debt financing decisions. Albeit borrowings may facilitate the firms for the time being but creates multiple threats for the firms, such as default risk. This study contributes to the existing literature; first, it expands the board gender diversity literature to examine the board gender diversity debt-taking decision. Secondly, the study examines the board effect on a firm's financial decision-making. Finally, the study will shed light on the call for diversity on the board for the betterment of firms. The firms facing weak corporate governance should see the result in their outcome by adding more females to their board as it will directly affect firm performance and debt financing.

The study organizes as follows; the following section explains the literature. Section three explains the methodology and data sources. The fourth section presents the testing of the hypothesis and findings of the study. The last section concludes the study along with limitations and future direction.

## Review of literature

Agency theory states that managers are opportunists and perform actions to maximize their wealth (Jensen and Meckling, 2019). Homogenous boards (Traditionally males), managers made risky investments through massive borrowings due to their overconfidence and poor monitoring system. In contrast, the literature suggests that female participation in the board tightens monitoring and improves financial decisions (Fields et al., 2012; Datta et al., 2021). This monitoring ability is perceived as women's risk averseness.

In contrast, Gender socialization theory (Dawson, 1997) states that males and females learn different roles, concerns, and values in childhood which develop different moral principles. These different moral principles played a vital role in their decision-making. Velthouse and Kandogan (2007) found that females are more sensitive to ethical concerns and less likely to be involved in security fraud. Cumming et al. (2015) concluded that firms with gender-diverse boards have fewer security frauds than homogenous gender boards. It shows that females' presence on the board is in the best interest of the stakeholders. The above theoretical debate encourages and supports the involvement of females in boardrooms.

Back in there was an immersed pressure for the betterment of the governance structure; some of the leading countries that followed these changes, such as Norway, imposed a law back in 2003 which demanded the public listed firms to fill the board position with at least 40% with the presence of women by 2008 (Ahern and Dittmar, 2012). Females at high posts are likely to differ from each other in terms of their perspectives and knowledge. In different roles, females seem to be at different scales in risk as compared to males, who seem more risk averse than males (Croson and Gneezy, 2009). Being in the boardroom, as per mass theory, if females are prominent in number; as a result, they ask more questions and board decisions are more productive with the decision making. Due to limited status and gender labeling, female directors may be treated as ordinary or unimportant in the boardroom, and their impact on corporate decisions is likely to be limited. So by changing the status of being “token” to “voice” the critical mass theory has a crucial weight in the corporate governing structure; this is because the female is now influencing the boardroom, and this impact should be more evident (Kristie, 2011).

The fall of meth about men driven the economies had been abolished after the end of financial crises back in 2008/09. Emerging economies like China have continually evolved their corporate governance policies so that firms and related parties can never default due to debt failure. China, the largest economy with a better and more recognized governance structure, provides an ideal setting to test the organizational level theories. In 2006, the China Securities Regulatory Commission (CSRC) urged the corporation to improve the corporate governance structure as a priority. As a result, a massive increase has been seen in the boardroom directors' duality role. Most of the analysis shows that executive post females act more cautiously than male executives regarding the corporation's financial or non-financial decisions (Adams and Funk, 2012).

The previous research had never fully answered China's firm performance and debt financing case. Corporate governance and policy making in China are prominently weaker than in the United States and other developing nations (Allen et al., 2005). So does diversity in the boardroom play a vital role? In China, most listed firms are state-owned enterprises, so the effect of diverse mass theory requires involvement from state owners (Government representatives). It was seen that the board gender diversity effect is positive and substantial in legal person control firms but inconsequential in state-controlled firms (Chen et al., 2006).

Prior studies showed that gender-diverse boards are associated with high performance and board fiduciary. Notably, females' presence in the board room is vital in highlighting and raising the critical issues considered unpalatable in homogenous (all-male) boards (McInerney-Lacombe et al., 2008). Usman et al. (2019) gender diverse board reduces opportunistic managerial behavior and improves asymmetric information, resulting in better performance and fewer default chances, lowering the debt cost. Zaid et al. (2020) argued that the presence of female directors tightens the board monitoring and effective strategic decisions. In the same line, Gul et al. (2011) argued that females are not overconfident as their counterpart males and do not make risky financial decisions. They always demand a high standard of assessment and validation of the manager's reports. An extensive board discussion and more information reduce the probability of bad decisions and prevent managers from investing in high-risk projects.

Back in there was an immersed pressure for the betterment of the governance structure; some of the leading countries that followed these changes, such as Norway, imposed a law back in 2003 which demanded the public listed firms to fill the board position with at least 40% with the presence of women by 2008 (Ahern and Dittmar, 2012). Females at high posts are likely to differ from each other in terms of their perspectives and knowledge. In different roles, females seem to be at different scales in risk compared to males, who seem more risk-averse than males (Croson and Gneezy, 2009). Being in the boardroom, as per mass theory, if females are prominent in number; as a result, they ask more questions and board decisions are more productive and well-driven.

Adams and Ferreira (2009) argued that female presence on the board improves the male board members' participation in the board meetings and involvement in different committees. Srinidhi et al. (2011) explained the gender-diverse board as females are not part of the old boys' network and show more independence and high moral conscience, which is necessary for effective oversight. Additionally, females have a low tolerance for opportunistic behavior, which harms stakeholders and lenders. In this study, our interest lies how a gender-diverse board made debt financing decisions. In the light of the above discussion, the study hypothesizes that,

*Gender-diverse boards are negatively associated with debt financing*.

Several studies in the literature highlight the importance of board independence on firm performance as they protect the investor's investment in the firms (Rashid, 2018; Shan, 2019; Al-Gamrh et al., 2020). Financing decisions of the firms are considered the board's strategic decisions, and independent boards make these decisions in favor of stakeholders. According to the agency theory, an independent board uses debt financing more effectively. Yazdanfar and Öhman (2015) examined the role of board characteristics and Swedish firm performance. They concluded that higher debt ratios in terms of short-term and long-term debt hurt financial performance and increased agency issues. In the same direction, Anderson et al. (2004) found that independent directors are associated with a lower yield spread among Standard and Poor 500 companies. Klein (2002) supports the Yazdanfar and Öhman (2015) findings that independent directors on the board are less likely to book abnormal accruals. The above studies showed that independence in the boardroom improves the firm's transparency and hence reduces the leverage of the firm. In the light of the above-stated literature, the study hypothesizes that,

*Board independence is negatively associated with the debt financing*.

## Data and methodology

The governance and debt financing data are taken from China Stock Market and Accounting Research Database (CSMAR) from 1991 to 2021. In line with (Prommin et al., 2014), some financial firms are excluded due to their special reporting requirements. Firms are taken from 1991 because China made substantial growth toward the governance codes and thus selected the sample to explore the influence of gender diversity on debt financing among Chinese firms. The study used unbalanced panel data. We employed the fixed effects regression suggested by the Durbin–Wu–Hausman test. Variables description and measurement are explained (see Table 1).

**Table 1:** Variables description and measurement.

**Variable**	**Name**	**Measurement**	**Sources**
**Independent variable**			
WOBF	Women board diversity	Women in the board/ Total board size	(CSMAR)
Women dummy	Women dummy	Dummy variable; equals 1 when there is at least one female on the board, 0 otherwise	Self-calculated
D1	Women dummy1	A dummy variable; equals 1 if a firm has one woman female on its board and 0 otherwise	Self-calculated
D2	Women dummy2	A dummy variable; equals 1 if a firm has at least two female directors on its board and 0 otherwise	Self-calculated
D3	Women dummy3	A dummy variable; equals 1 if a firm has at least three female directors on its board and 0 otherwise	Self-calculated
BIND	Board independence	Number of independent directors on the board	(CSMAR)
**Dependent variable**			
GL	Total gross debt	Sum of short-term debt and long-term debt	(CSMAR)
STL	Short term loan	Sum of all loans having maturity of 1 year	(CSMAR)
LTL	Long term loan	Sum of all loans having maturity of more 1 year	(CSMAR)
**Control variables**			
TA	Total assets	Sum of all current assets and non-current assets	(CSMAR)
CFPS	Cash flow per share	Cash flow earned in a given reporting period/ total number of shares outstanding	(CSMAR)
BS	Board size	Number of board of directors in the board	(CSMAR)
LR	Debt to total market capitalization	Total debt / (debt + equity + minority interest + preferred stock)	(CSMAR)

The descriptive statistics showed that the average board size among Chinese companies is 10 while the maximum is 26 members. The presence of females found in all the companies, as evident from the mean of female's board in the above Table 2, is obvious due to the corporate governance codes companies are required to involve female members on the board. The healthiest signal for the Chinese companies is that over time, they increased the involvement of females on the board as the maximum number of females on a board was found 9. The mean of the short-term and long-term loans represents that Chinese companies are using debt financing significantly. The high debt service coverage ratio shows that Chinese firms produced significant profits to manage their interest payments.

**Table 2:** Descriptive statistics.

**Variables**	**Obs**	**Mean**	**Std. Dev**.	**Min**	**Max**	**p1**	**p99**	**Skew**.	**Kurt**.
Number of directors	53,031	9.424	2.45	0	26	5	17	1.135	5.846
Including number of females	53,031	1.254	1.162	0	9	0	5	1.035	4.358
Including number of independent directors	53,031	3.255	1.253	0	13	0	7	0.207	6.15
LTD	30,476	19.196	2.252	4.061	26.641	13.266	24.624	−0.129	3.647
STD	42,178	19.453	1.799	0.693	25.901	14.509	23.647	−0.372	4.352
GL	44,489	19.843	1.989	9.183	26.84	14.509	24.663	−0.231	3.979
LR	44,462	0.221	1.315	0	213.046	0.001	0.689	140.964	21,141.851
TA	44,462	21.937	1.392	12.27	29.811	19.286	26.166	0.822	4.563
CFPS	44,595	0.001	0.064	−0.27	8.8	0	0	109.261	12,909.93

To handle multi-collinearity problems, the correlation among dependent and independent variables is calculated in Table 3. The results showed that all the correlation values are within the acceptable range (0.70) for Liu et al. (2014). There is a significant negative association between the percentage of females on the board and short-term debt as (−0.082^*^) and a similar foundation for the long-term debt (−0.067^*^) while the board size found positively correlated with the debt financing either short-term (0.161^*^) or long-term loan (0.142^*^). The following table shows the comparison of loan-taking behavior among the firms having females on board and those having male-dominated boards.

**Table 3:** Correlation matrix.

**Variables**	**(1)**	**(2)**	**(3)**	**(4)**	**(5)**	**(6)**	**(7)**	**(8)**
(1) wobf	1.000							
(2) BS	−0.077^*^	1.000						
(3) BID	0.037^*^	0.558^*^	1.000					
(4) LR	−0.002	0.004	0.001	1.000				
(5) TA	−0.067^*^	0.206^*^	0.289^*^	−0.050^*^	1.000			
(6) LTD	−0.067^*^	0.142^*^	0.202^*^	0.263^*^	0.709^*^	1.000		
(7) STD	−0.082^*^	0.161^*^	0.178^*^	0.057^*^	0.675^*^	0.567^*^	1.000	
(8) CFPS	−0.003	−0.003	0.002	−0.001	−0.007	−0.006	−0.009	1.000

^***^p < 0.01,

^**^p < 0.05,

* p < 0.1.

## Results and discussion

The results in Table 4 showed that the debt-taking behavior of firms having females on board significantly differs from those with no females on the board. This difference exists in both types of debt in short-term and long-term debt financing. Results show that female participation in the board does influence the company's debt financing decisions.

**Table 4:** Two-sample *t* test with equal variances.

	**obs1**	**obs2**	**Mean1**	**Mean2**	**t value**	***p*** **Value**
LTD by womend: 0 1	9,294	21,182	19.272	19.163	3.9	0
STD by womend: 0 1	12,658	29,520	19.532	19.42	5.85	0

We employed the Ordinary Least Square (OLS) and Fixed Effects (FE), and Random Effects (RE) regressions to test the hypothesis. The human test supports the Fixed Effects, and the results are presented in Table 5.

**Table 5:** Gender diversity and debt financing.

	**(1)**	**(2)**	**(3)**
	**LTD**	**STD**	**GL**
Wobf	1.103^***^	0.369^***^	0.501^***^
	(0.1)	(0.072)	(0.073)
LR	1.815^***^	0.03^***^	0.034^***^
	(0.052)	(0.005)	(0.005)
BS	−0.68^***^	−0.398^***^	−0.492^***^
	(0.056)	(0.041)	(0.041)
BID	0.287^***^	0.198^***^	0.231^***^
	(0.009)	(0.007)	(0.007)
TA	0^***^	0^***^	0^***^
	(0)	(0)	(0)
CFPS	−0.307		
	(0.197)		
_cons	19.109^***^	19.589^***^	20.052^***^
	(0.113)	(0.08)	(0.082)
Observations	30,362	42,064	44,332
R-squared	0.102	0.043	0.054
Industry dummies	Yes	Yes	Yes
Year dummies	Yes	Yes	Yes

Standard errors are in parentheses

*** p < 0.01,

^**^ p < 0.05,

^*^ p < 0.1.

The results revealed that firms having a female presence on the board are positively associated with the debt-taking behavior of Chinese listed firms. The results rejected the glass ceiling view and supported Babalos et al. (2015) findings that females are taking the risk and rely on debt financing when they believe the firms have potential growth opportunities. The results showed that female presence is a positive influence the short-term debt (0.369^***^) and long-term debt (1.103^***^), and gross loans (0.501^***^). The findings support our first hypothesis that the presence of females on board increases debt financing. The independent director's role in the company's essential decisions is always worthwhile, and debt financing has equal importance. The results revealed that board independence significantly influences the company's debt-taking decisions. The coefficient of the independent board for short-term financing and long-term financing are (0.287^***^) and (0.198^***^), respectively. The findings contradict Yazdanfar and Öhman (2015) state that independent board directors are less likely to book abnormal accruals. We employed the critical mass theory to explore the role of diversity and robust our findings. Kristie (2011) concludes the theory by citing that “one is a token, two is a presence, and three is the voice.” The literature suggests that female presence is only fruitful when the number of females on board is greater than one (Kramer et al., 2006). The results of critical mass are presented in Table 6.

**Table 6:** Fixed effect–critical mass.

	**(1)**	**(2)**	**(3)**
	**LTD**	**STD**	**GL**
WD1	−0.04	0.008	−0.01
	(0.026)	(0.019)	(0.019)
WD2	−0.012	−0.038	0
	(0.037)	(0.027)	(0.027)
ED3	0.171^***^	0.099^***^	0.093^***^
	(0.038)	(0.027)	(0.028)
LR	1.808^***^	0.03^***^	0.034^***^
	(0.052)	(0.005)	(0.005)
BS	−0.735^***^	−0.417^***^	−0.514^***^
	(0.056)	(0.041)	(0.041)
BID	0.293^***^	0.199^***^	0.233^***^
	(0.009)	(0.007)	(0.007)
TA	0^***^	0^***^	0^***^
	(0)	(0)	(0)
CFPS	−0.316		
	(0.198)		
_cons	19.263^***^	19.628^***^	20.103^***^
	(0.112)	(0.08)	(0.081)
Observations	30,362	42,064	44,332
R-squared	0.1	0.043	0.054
Industry dummies	Yes	Yes	Yes
Year dummies	Yes	Yes	Yes

Standard errors are in parentheses

*** p < 0.01,

^**^ p < 0.05,

^*^ p < 0.1.

To apply this assumption, the study followed Liu et al. (2014) and created three dummies for the female's presence in the board room. D1 equals 1 if there is only one female; otherwise, 0. D2 equals 1 if two females are on the board, 0 otherwise. D3 equals 1 if at least three females are in the board room 0 otherwise. The findings confirm that firms having only one female in the board room, their relationship with short-term financing is negative (−0.04) but insignificant. For the long-term loan it is positive but (0.008) and insignificant.

The coefficient of D2, which is (−0.012) for long-term financing and (−0.38) for the short-term, shows that the diversity of up to two females on the board did not give fruit similar as proposed by the critical mass theory. The coefficient of D3, (0.171^***^) for short-term and (0.099^***^) for long-term loans, confirms that diversity pays off when the percentage of females on the board is reasonable to create their voice and influence the decisions. The results confirm Liu et al. (2014) summary of mass theory, in which they summarized that female more than three are the voice of females on the board. These further highlight the importance of board diversity that simply diversity may not benefit firms until it gains a voice in the board that is at least three females, as suggested by Liu et al. (2014). The results further strengthen our hypothesis that gender diversity will generally influence the debt financing decisions but will significantly influence the debt financing decision with their good presence on the board.

## Conclusion

Previous studies on lottery and gambling have distinguished men and women and tagged women as more risk averse than their counterparts men. Our study provides unique findings that firms' debt financing increases susceptibility to various risks, such as liquidity risk. Default risk is positively associated with the female's presence in board rooms of the Chinese listed firms. The results support the recent call for the involvement of females on the company's board and involvement in the company's decisions as they are risk averse. The study further highlights the need for female participation on the board by studying the mass theory and recommends that more females on the board are more beneficial for firms. The study has several theoretical contributions. First, it enhances the gender diversity literature by arguing and contracting the stereo typo view that females are risk averse. Secondly, the study extends the gender diversity literature by highlighting that females' presence to fulfill regulator requirements is not the optimal choice. Still, the involvement of more females is more likely to make better decisions for the firms. Lastly, the study found that female presence is not rigid on growth-oriented loans but supports short-term loans. The study has several practical implications. Firstly, the study facilitates the stakeholder that they must value the firms and parks their investment where the female's presence is high. The study is also helpful for the regulators to improve and update the diversity codes by increasing female presence on the board to get better diversity results. The study has a few limitations. The study only used the Chinese listed data, and it would be hard to generalize the results with the developed countries. It would be difficult to imply the same results with small economies compared to China. Further studies may do comparative studies within the region and with the developed countries. Further studies may take other diversity along with gender diversity to explore diversity fruits.

## Data availability statement

The data analyzed in this study is subject to the following licenses/restrictions: we used the data from CSMAR and only available to the official students of the university and we cannot share this data to anyone else. As this is purchased database requests to access these datasets should be directed to https://cn.gtadata.com.

## Author contributions

XS originates the idea and is responsible for the analysis part. MA wrote the initial draft. KT proofread the document and validated the analysis. HZ was responsible for the methodology section, software, and resources. All authors contributed to the article and approved the submitted version.

## Funding

This work is partially supported by National Natural Sciences Foundation of China Grant Number (72172031).

## Conflict of interest

The authors declare that the research was conducted in the absence of any commercial or financial relationships that could be construed as a potential conflict of interest.

## Publisher's note

All claims expressed in this article are solely those of the authors and do not necessarily represent those of their affiliated organizations, or those of the publisher, the editors and the reviewers. Any product that may be evaluated in this article, or claim that may be made by its manufacturer, is not guaranteed or endorsed by the publisher.
